# Supplementary material for: Topography-induced large-scale antiparallel collective migration in vascular endothelium
Source: Nat Commun. 2022 May 19;13:2797. doi: 10.1038/s41467-022-30488-0 (PMC9120158; doi:10.1038/s41467-022-30488-0)
Supplement: Supplementary file 1 — Supplementary Information [file 41467_2022_30488_MOESM1_ESM.pdf]

Supplementary Information

Topography-induced large-scale antiparallel collective migration in vascular endothelium

Claire Leclech[1], David Gonzalez-Rodriguez[2], Aurélien Villedieu[3], Thévy Lok[1],

Anne-Marie Déplanche[4], Abdul I Barakat[1*]

[1]LadHyX, CNRS, Ecole Polytechnique, Institut Polytechnique de Paris, Palaiseau, France.

[2]Université de Lorraine, LCP-A2MC, F-57000, Metz, France.

[3]Institut Curie, Université PSL, Sorbonne Université, CNRS UMR 3215, Inserm U934, Genetics and Developmental Biology, 75005 Paris, France.

[4]University of Nantes, LS2N UMR 6004, 1 rue de la Nöe - BP 9210 Nantes 44321.

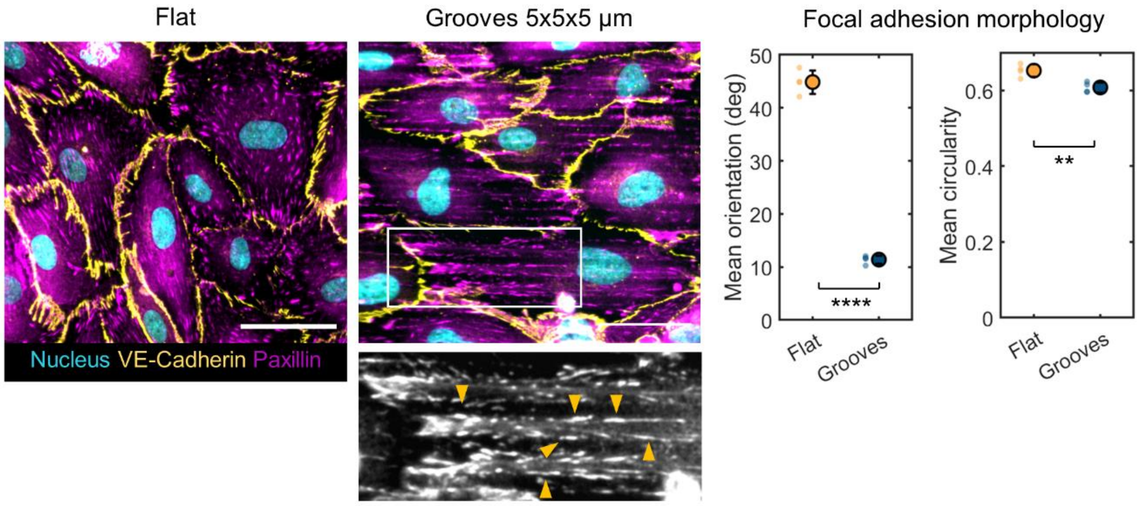

Supplementary Figure 1: Effect of microgrooves on focal adhesion morphology. Immunostaining for VE-cadherin and paxillin in a HUVEC monolayer on a flat surface or on 5 x 5 x 5 µm (w x s x d) grooves. Yellow arrowheads point to the localization of focal adhesions (FAs) along the ridge edges. Scale bar 50 µm. FA morphology was analyzed using a custom-made FIJI macro that segments FAs using different steps of filtering and thresholding. Circularity is defined as the FA minor-to-major axis ratio. Unpaired t-test, n=3 independent experiments (**** p<0.0001, ** p<0.007), and error bars represent standard deviations. Source data are provided as a Source Data file.

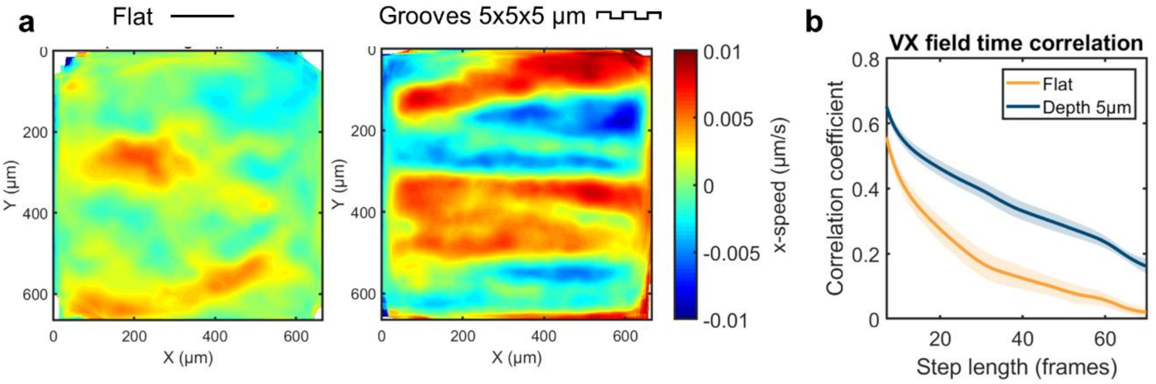

Supplementary Figure 2: Establishment of the stream pattern on microgroove substrates. a, Time-averaged x-direction velocity fields on flat surfaces and on 5 x 5 x 5 µm (w x s x d) grooves. **b**, Quantification of the time correlation length: the Pearson correlation coefficient is calculated for pairs of frames increasingly distant in time. Error bars represent SEM, n=3 independent experiments. Source data are provided as a Source Data file.

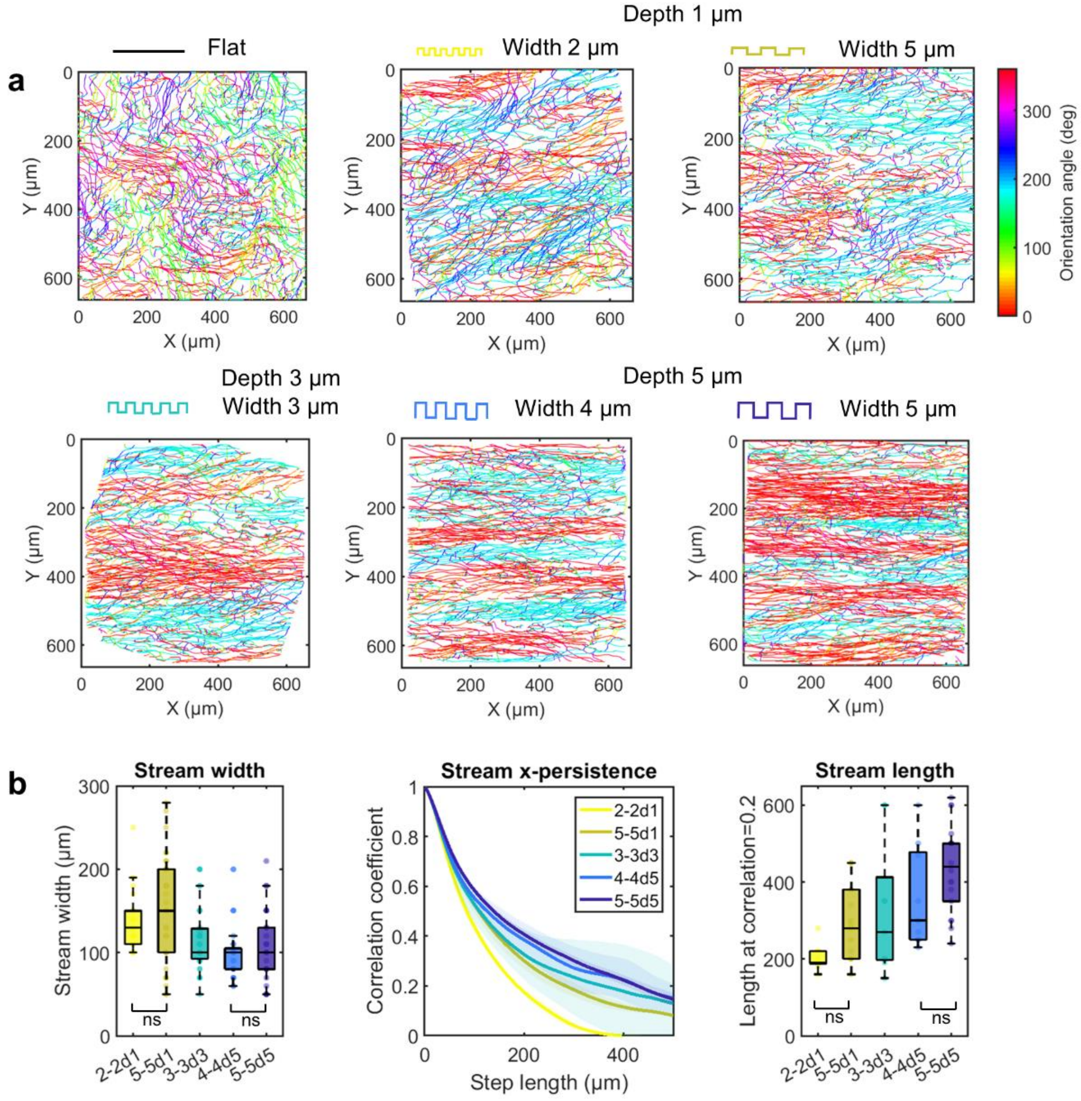

Supplementary Figure 3: Influence of groove width on the spatial features of the streams. a, Accumulated cell trajectories after 24 h of migration for flat control substrates and for microgroove substrates of different dimensions: w=s=2 μm d=1 μm (2-2d1), w=s=5 μm d=1 μm (5-5d1), w=s=3 μm d=3 μm (3-3d3), w=s=4 μm d=5 μm (4-4d5), w=s=5 μm d=5 μm (5-5d5). Trajectories are color-coded for the orientation angle of each displacement vector. **b**, Quantification of the spatial features of the streams with the same method as explained in the main text. One-way ANOVA, Dunn's/Fisher's post-test, n=3 independent experiments. Source data are provided as a Source Data file.

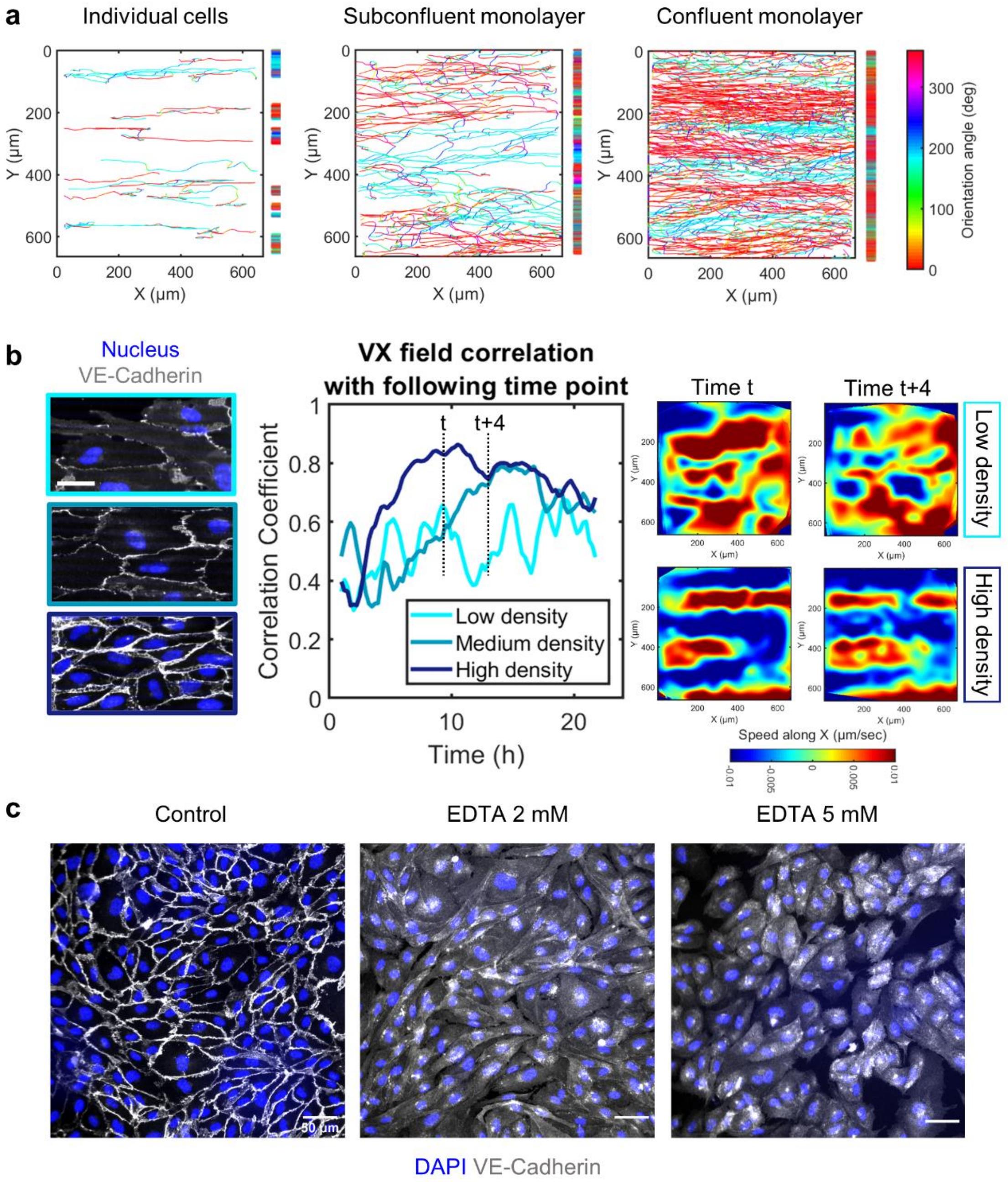

Supplementary Figure 4: Emergence of cell streams with increasing cell density. a, Accumulated cell trajectories after 24 h of migration for individual cells, low-density monolayer, and high-density monolayer on grooves of width, spacing, and depth of 5 µm. Trajectories are color-coded for the orientation angle of each displacement vector, and vertical bars represent the projected trajectories on the y-axis. **b**, Time-averaged x-direction velocity field for monolayers of different densities and immunostaining for VE-cadherin showing the morphology of cell-cell junctions for the approximate same cell density. Scale bar, 30 µm. The fields of view (FOVs) correspond to movie3. The graph shows for these 3 FOVs the time evolution of the correlation between the V_x field at time t and at t+1 and illustrates the dynamic evolution of the collective pattern of motion. **c**, Immunostaining for VE-cadherin and DAPI on HUVEC monolayers after 48 h of culture on flat surfaces for control, 2 mM EDTA, or 5 mM EDTA treatment (24 h). Source data are provided as a Source Data file.

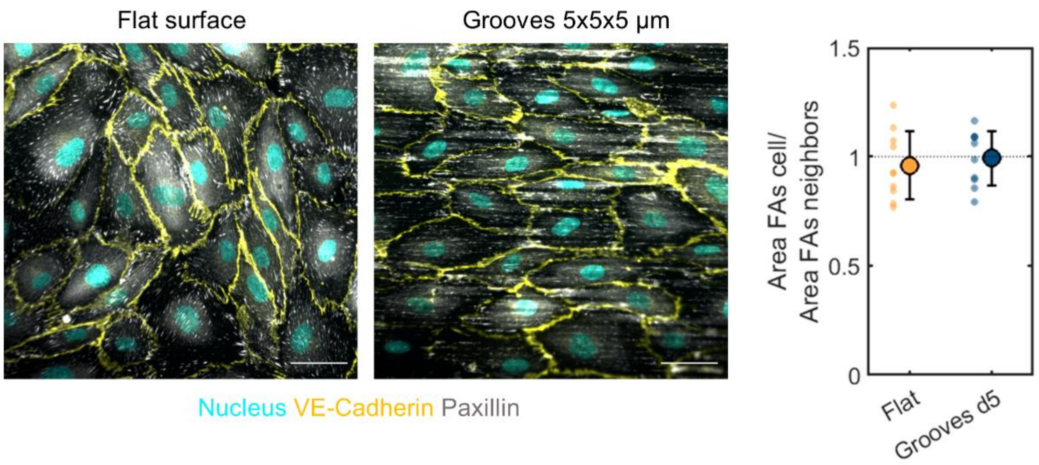

Supplementary Figure 5: Focal adhesion area within the monolayer. Immunostaining for VE-cadherin and paxillin in a HUVEC monolayer on a flat surface or on 5 x 5 x 5 µm (w x s x d) grooves. Scale bar 50 µm. The mean FA area within one cell was compared to the mean area of FAs in its neighbors. N = 10 cells, error bars represent standard deviations. Source data are provided as a Source Data file.

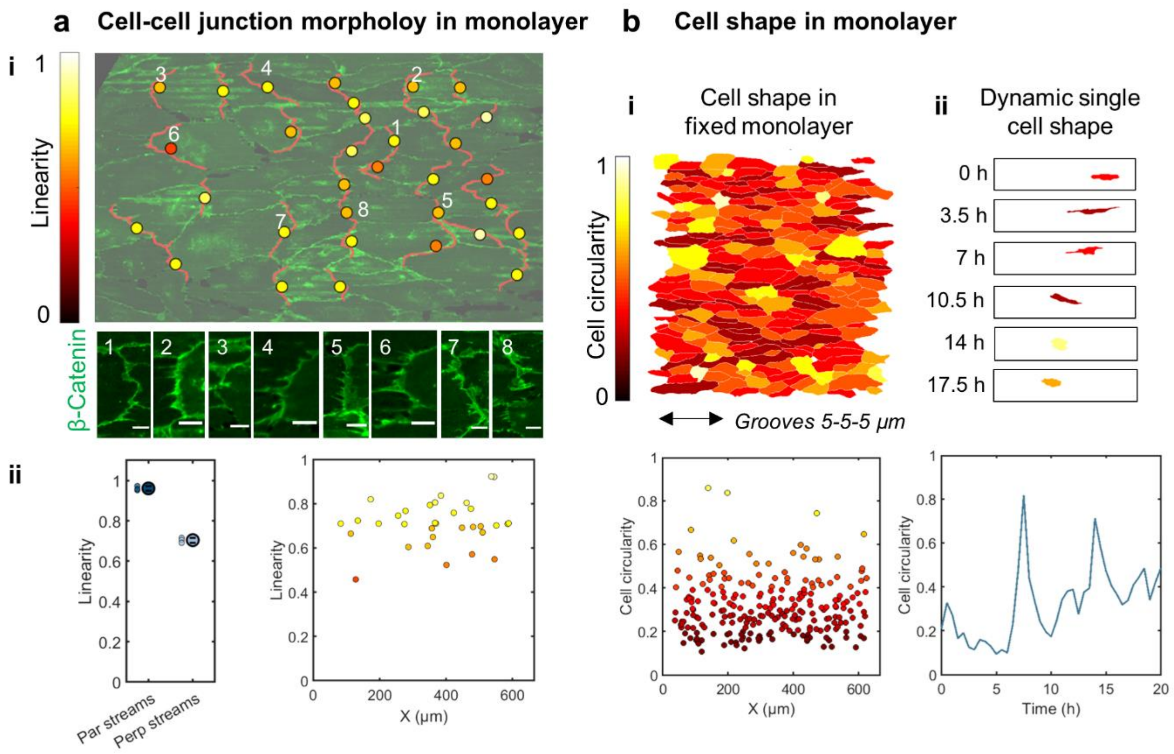

Supplementary Figure 6: Cell-cell junction morphology and cell shape within the monolayer. a, (i) HUVEC monolayer on 5 x 5 x 5 µm (w x s x d) grooves immunostained for β-catenin to enable cell-cell junction morphology analysis. Previous studies have described mature linear junctions vs. "zipper-like" or "finger-like" junctions that are under tension and that are typically present between leader and follower cells[1–3]. (ii) Left: linearity of junctions in the direction of the grooves/streams ("Par streams") or perpendicular to the grooves/streams ("Perp streams"), quantified by the ratio of length of line connecting junction-end points to the actual length of junction. Junctions are linear junctions in the groove/stream direction and zipper-like perpendicular to the grooves/streams, confirming the general direction of traction in the monolayer. N=3 fields of view, error bars represent standard deviations. Right: linearity values of zipper-like junctions perpendicular to grooves (see insets, scale bars 10 µm) are similar across the monolayer, suggesting minimal heterogeneity in traction forces among cells. **b**, (i) Cell shape in fixed monolayers analyzed using a custom-made Matlab code that automatically calculates cell shape parameters from cell outlines segmented from cell-cell junction staining. Note that although cells within the monolayer exhibit different levels of elongation, as quantified by the cell circularity (minor-to-major axis ratio), no clustering of elongation is present. This is unlike leader-follower systems where actively pulling leader cells are typically more elongated than follower cells[4]. (ii) Shape of a single cells at different times extracted from recordings of GFP VE-cadherin HUVECs (gift from Dr. Laurent Muller, Collège de France, Paris, France) on 5 x 5 x 5 µm (w x s x d) grooves. Single cells can, in the span of hours, explore the entire range of instantaneous cell elongations found within the monolayer, suggesting rapidly evolving patterns of cellular forces. Source data are provided as a Source Data file.

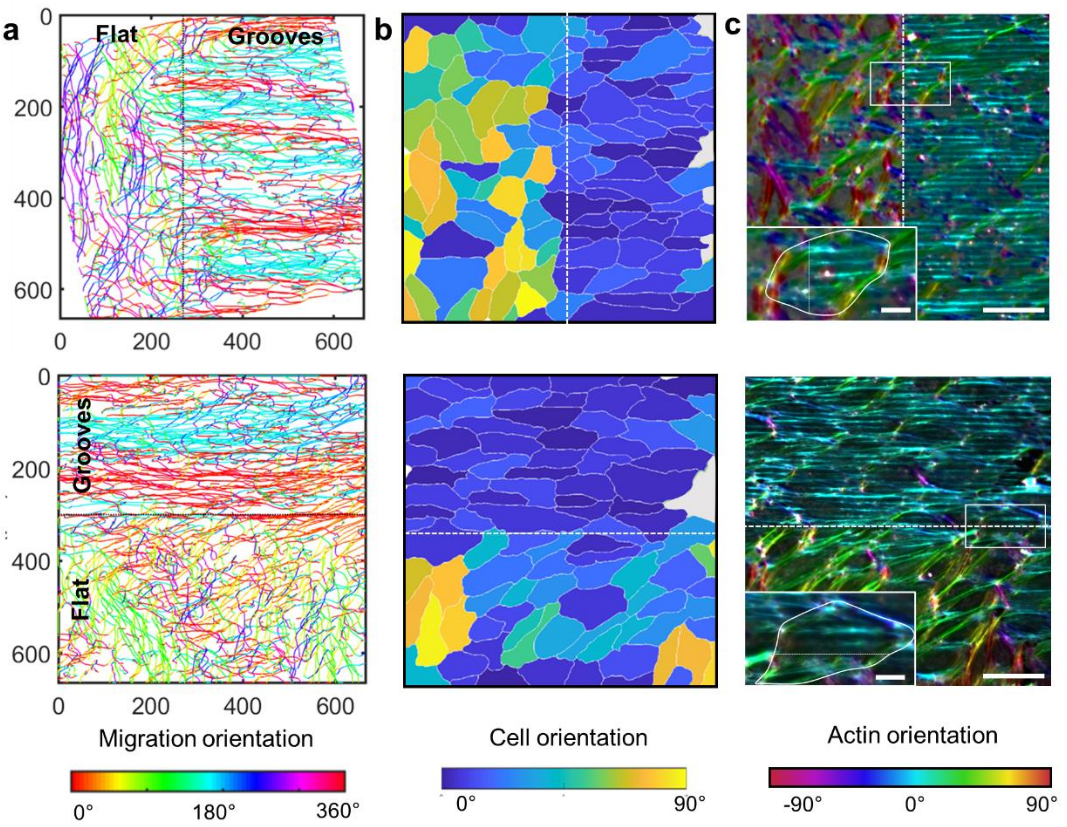

Supplementary Figure 7: Analysis of the monolayer at the border between grooves and flat surface. a, Accumulated cell trajectories after 24 h of migration at the border between 5 x 5 x 5 µm (w x s x d) grooves and flat surface, color-coded for the orientation of each displacement vector. **b**, Cell orientation angle at the border between 5 x 5 x 5 µm (w x s x d) grooves and flat surface. Cells in fixed monolayers were analyzed using a custom-made Matlab code that automatically calculates cell shape parameters from cell outlines segmented from cell-cell junction staining. **c**, Actin organization at the border between 5 x 5 x 5 µm (w x s x d) grooves and flat surface, color-coded for the orientation of actin bundles (using Orientation J plugin, FIJI). Insets (white rectangles) show actin organization in cells that are positioned partly on grooves and partly on flat surface. Scale bars 100 µm, 25 µm for insets.

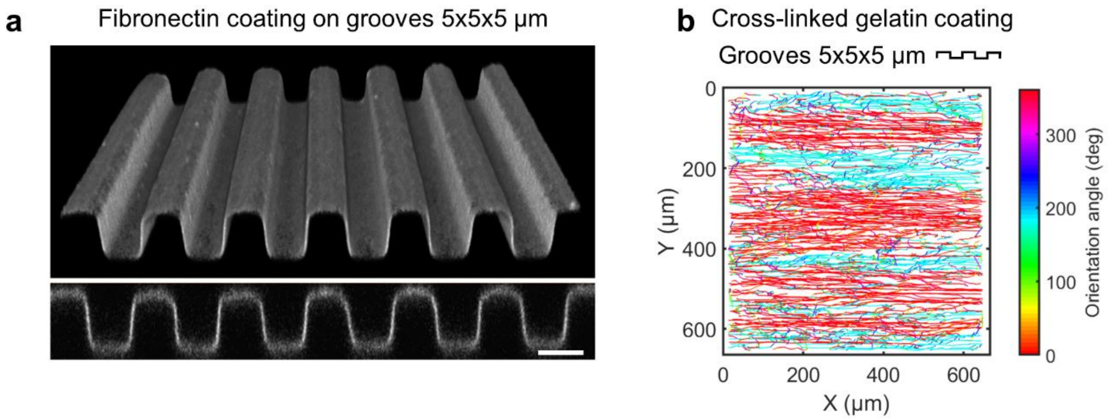

Supplementary Figure 8: Influence of substrate coating on stream formation. a, Confocal 3D reconstruction and cross-section of fluorescent fibrinogen showing homogeneity of fibronectin coating on 5 x 5 x 5 µm (w x s x d) grooves. Scale bar 5 µm. **b**, Accumulated cell trajectories after 24 h of HUVEC migration on 5 x 5 x 5 µm (w x s x d) grooves coated with cross-linked gelatin, color-coded for the orientation angle of each displacement vector.

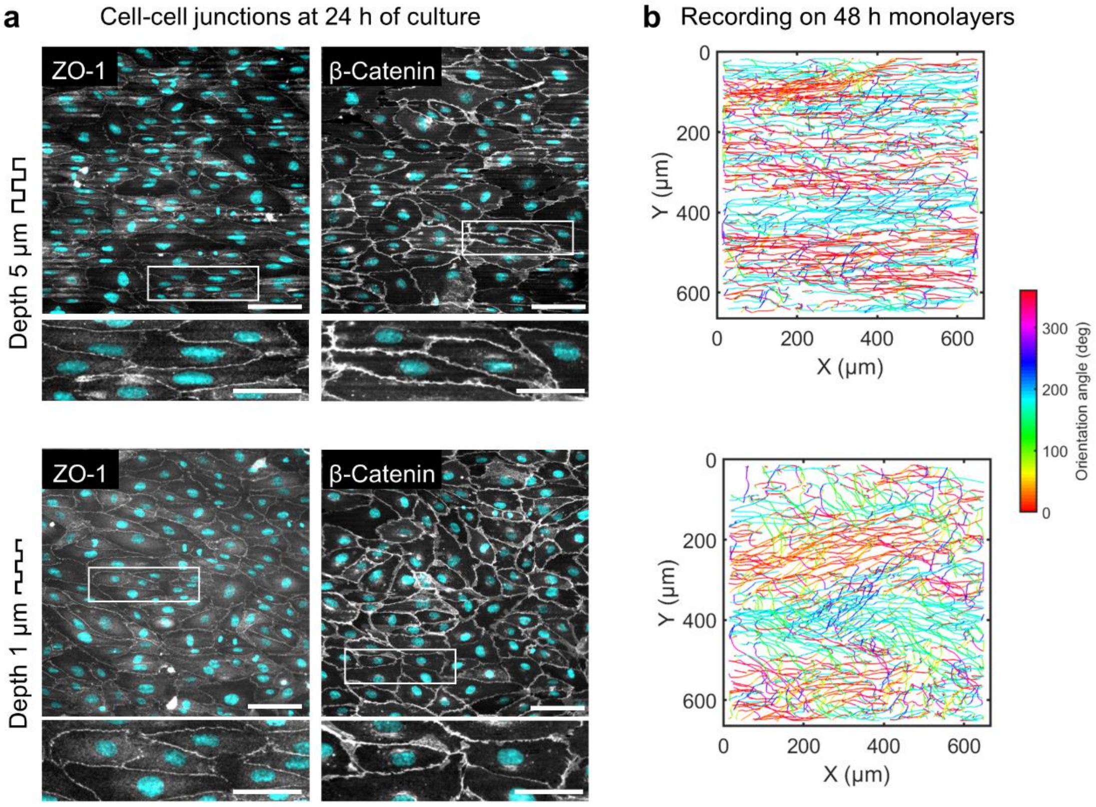

Supplementary Figure 9: Influence of time in culture on cell-cell junctions and stream formation. a, Immunostaining for the cell-cell junction proteins ZO1 and β-catenin in HUVECs after 24 h of culture on 5 µm- or 1 µm-deep grooves. Zoom-ins are shown in insets (white rectangles). Scale bars 100 µm, 50 µm for insets. **b**, Accumulated cell trajectories of HUVECs on 5 µm- or 1 µm-deep grooves recorded after 48 h of culture, color-coded for the orientation angle of each displacement vector.

Supplementary note: Theoretical modeling

We explain the emergence of endothelial cell streams by an active fluid model inspired by that proposed by Duclos *et al.*[5]. Duclos *et al.* applied the model of Voituriez *et al.*[6] based on active gel theory[7] to describe spontaneous flows in confined cell sheets. They explained the emergence of global directional motion of otherwise non-migrating cells in a confined stripe with cells advancing in opposite directions at the two ends of the stripe. To account for the specificities of our experiment, the model differs from that of Duclos *et al.* in that an additional term in the free energy equation is added to account for the tendency of cells to orient along the ridges.

Active fluid equations. We consider a 2D coordinate system (x,y) with the x-direction aligned with the ridges and the y-direction perpendicular to them. Endothelial cells are treated as active nematic particles whose orientation is characterized by the unit director field $\boldsymbol{p} = (\cos\theta, \sin\theta)$, with θ being the angle between the cell orientation and the ridges. Because endothelial cells are able to change their direction of motion without rotating the cell body, we model them as nematic particles, a hypothesis also made by Duclos *et al.*[5]. Moreover, our experiments show that endothelial cells on microgrooves typically change direction of migration over a time scale on the order of 1-2 hours, whereas the establishment of the streams occurs over times of about one day. This separation of time scales further supports using the nematic cell description for the purpose of describing stream formation. Actomyosin cell activity induces a contractile (or extensile) force dipole acting on each cell. The force per unit length of the cell is given by $\zeta\Delta\mu$, where $\Delta\mu$ is the free energy produced by nutrient consumption and ζ is the reactive coefficient as defined in active gel theory[7], whose sign indicates whether active forces are contractile (ζ <0) or extensile (ζ >0). Note that the term $\zeta\Delta\mu$ is a force dipole. Therefore, the associated net force acting on a single cell is zero, and

this term does not lead to single cell locomotion. It is indeed notable that single cell locomotion is not a necessary ingredient for the formation of the streams described here.

Effective free energy. The effective free energy of the director field in two dimensions is given by

$$\mathcal{F} = \int \left(\frac{K_1}{2} (\nabla \cdot \boldsymbol{p})^2 + \frac{K_3}{2} (\nabla \times \boldsymbol{p})^2 + \frac{\alpha}{2} p_y^2 \right). \tag{1}$$

The first two terms are the classic formulation of the effective free energy in active gels, where K_1 and K_3 are the splay and bend Frank constants, respectively. As in Duclos *et al.*[5], we assume $K_1 = K_3 = K$. The third term is a new term that we introduce to penalize cell orientation across the ridges. The coefficient α is a measure of the tendency of the cells to align with the ridges that is expected to depend on the ridge height. By using the Einstein relation between diffusion and active thermal agitation, this coefficient can be estimated as $\alpha \approx \xi_\mathrm{x} D_\mathrm{t}/\sigma_\theta^2$, where ξ_x is the substrate friction coefficient, D_t the translational diffusion, and σ_θ the standard deviation of cell orientation angles. We have measured for our cells $D_\mathrm{t} \approx 10$ μm^2/min, which leads us to estimate α to be in the range 10^{-3}–10^{-2} J/m^2, depending on the value of σ_θ which is determined by the groove geometry. Importantly, the estimate of α, which is determined by groove geometry, is insensitive to cell density, as we obtain similar estimates in experiments with densities ranging from isolated to confluent cells (Supplementary Table 1). This validates our application of the Einstein relation to an out-of-equilibrium system, as it has been shown that such application to active matter may remain formally valid in the dilute regime[8].

Supplementary Table 1: Mean standard deviations of cell displacement orientation angles (degrees) for different groove dimensions and cell densities.

	Grooves 5x5x1 μm	Grooves 5x5x5 μm
Monolayer	38.1	32.2
Individual cells	38.3	30.9

Constitutive relations. The linearized constitutive relations for an active nematic fluid in two dimensions relate the stress $\boldsymbol{\sigma}$ to the cell orientation $\boldsymbol{p}$ as well as the variations in cell orientation to the shear stress. They are:

$$\sigma_{\alpha\beta} = 2\eta u_{\alpha\beta} - \zeta\Delta\mu\, p_\alpha p_\beta + \frac{\nu}{2}\left(p_\alpha h_\beta + p_\beta h_\alpha\right) + \frac{1}{2}\left(p_\alpha h_\beta - p_\beta h_\alpha\right) - P\delta_{\alpha\beta}, \quad (2)$$

$$\partial_t p_\alpha + v_\beta \partial_\beta p_\alpha + \omega_{\alpha\beta} p_\beta = \frac{h_\alpha}{\gamma} - \nu u_{\alpha\beta} p_\beta. \quad (3)$$

Here, α and β are indices that stand for the directions x or y, with a repeated index implying summation over all values of the indices, as per Einstein's convention. $\sigma_{\alpha\beta}$ is the stress tensor, $u_{\alpha\beta} = (\partial_\alpha v_\beta + \partial_\beta v_\alpha)/2$ is the shear rate, with v_α the velocity component, and $\omega_{\alpha\beta} = (\partial_\alpha v_\beta - \partial_\beta v_\alpha)/2$ is the vorticity tensor. P is a Lagrange multiplier, related to the pressure, that enforces incompressibility, i.e., $\partial_\beta v_\beta = 0$. $h_\alpha = -\partial\mathcal{F}/\partial p_\alpha$ is the so-called orientational field. η and γ are the shear and rotational viscosities, respectively, arising from both cell mechanical properties and cell-cell interactions. υ is the flow alignment parameter, which controls how mechanical stress determines cell orientation.

Force balance. The force balance equation, assuming negligible inertia, reads

$$\partial_\beta \sigma_{\alpha\beta} = \xi_\alpha v_\alpha, \quad (4)$$

where ξ_α is the friction coefficient along the α-direction. Duclos *et al.* concluded that substrate friction did not significantly change the physics of the phenomenon they considered. Here we will show that substrate friction is needed to explain the emergence of periodic cell streams.

Simplification of the equations. We seek a quasi-1D solution to the problem defined by Eqs. (1-4) by assuming the solution to be uniform along x. Moreover, we assume that the velocity

along the ridges v_x dominates the transverse velocity v_y, which we neglect. We express the orientational field by its components parallel and perpendicular to the director, $h_\perp$ and $h_\parallel$. We obtain:

$$h_\perp = \gamma \left[\frac{(\nu \cos 2\theta + 1)}{2} \frac{\partial v_x}{\partial y} + \frac{\partial \theta}{\partial t} \right], \tag{5}$$

$$h_\parallel = \frac{\gamma\, \nu \sin 2\theta}{2} \frac{\partial v_x}{\partial y}. \tag{6}$$

Next, we linearize the force balance equation in the *x*-direction, Eq. (4), by assuming $\theta \ll 1$, to obtain:

$$\left(\eta + \frac{\gamma(1+\nu)^2}{4} \right) \frac{\partial^2 v_x}{\partial y^2} - \zeta \Delta\mu \frac{\partial \theta}{\partial y} + \frac{\gamma(1+\nu)}{2} \frac{\partial^2 \theta}{\partial t \partial y} = \xi_x v_x. \tag{7}$$

By using Eq. (5) and the relationship between the orientational field and the effective free energy, $h_\alpha = -\partial \mathcal{F} / \partial p_\alpha$, we obtain:

$$K \frac{\partial^2 \theta}{\partial y^2} - \alpha\theta = \frac{\gamma(1+\nu)}{2} \frac{\partial v_x}{\partial y} + \gamma \frac{\partial \theta}{\partial t}. \tag{8}$$

Eqs. (7) and (8) constitute a system of two ODEs with two unknowns: $v_x(y,t)$ and $\theta(y,t)$.

Linear stability analysis. We perform a linear stability analysis of the system formed by Eqs. (7) and (8) around a steady state where cells would all be immobile and aligned with the ridges, $\theta_{eq}(y)$=0, and $v_{x,eq}(y)$=0. We are specifically interested in finding the range of wavelengths (k_{min}, k_{max}) of the unstable modes, as well as the wavelength k_{*0} of the fastest growing mode, which will indicate the expected spatial periodicity of the flow profile at the onset of a perturbation. The linear stability analysis leads to the following dispersion relationship between the rate of growth σ and the wavenumber *k*:

$$\sigma = -\frac{(\alpha + Kk^2)}{\gamma}\left(1 + \frac{\gamma(1+\nu)^2k^2}{4(\xi_x + \eta k^2)}\right) - \frac{\zeta\Delta\mu(1+\nu)k^2}{2(\xi_x + \eta k^2)}. \tag{9}$$

In the limit α=0, this equation is identical to that obtained by Duclos *et al.* in the presence of substrate friction. Unstable modes can arise if $\zeta\Delta\mu(1+\nu) < 0$, for example in the case of contractile cells ($\zeta\Delta\mu < 0$). By solving the quadratic equation in k obtained by imposing σ = 0, we obtain the range of unstable wavenumbers ($k_{\min}$, $k_{\max}$). Based on the parameter estimates presented in Supplementary Table 2 below, the smallest possible stream width is $W_{\min}$ = $\pi/k_{\max} \approx 6$ µm, thinner than a single cell, which practically means that no lower bound on the stream width is imposed by the theory. Considering the dominant terms, the maximum width $W_{\max} = \pi/k_{\min}$ is given by

$$W_{\max} \approx \frac{\pi}{\sqrt{\xi_x}}\left[\frac{(1+\nu)}{2\alpha}\gamma(-\zeta\Delta\mu) - \eta - \frac{\gamma(1+\nu)^2}{4}\right]^{1/2}. \tag{10}$$

e At the onset of the perturbation, the fastest-growing wavenumber is:

$$k_{*0} = -\frac{\xi_x}{\eta} + \sqrt{\left(\frac{\xi_x}{\eta}\right)^2 + \frac{1}{l^4}}, \tag{11}$$

where

$$l^4 = \frac{K\eta\left[\frac{4\eta}{\gamma} + (1+\nu)^2\right]}{\xi_x\left[2(-\zeta\Delta\mu)(1+\nu) - \alpha(1+\nu)^2 - \frac{4K\xi_x}{\gamma}\right]}. \tag{12}$$

For k_{*0} to exist, the term in square brackets in the denominator of the right-hand side of Eq. (12) must be positive, which requires $2(-\zeta\Delta\mu)(1+\nu) > \alpha(1+\nu)^2$, since according to our parameter estimates (Supplementary Table 2), the third term in the denominator containing K is small compared to the other two. Thus, we assume the bracketed term in the denominator to be of the order of the active contractile term.

Supplementary Table 2: Estimates of model parameters.

Symbol	Meaning	Value	Units
$\lvert -\zeta\Delta\mu \rvert$	Cell contractility	10^{-2}	Pa·m
η	2D shear viscosity	10	Pa·s·m
γ	2D rotational viscosity	10	Pa·s·m
$\lvert 1+\nu \rvert$	Flow alignment parameter	1	
ξ_x	Substrate friction coefficient	10^{10}	Pa·s·m^{-1}
K	Frank constant	10^{-14}	Pa·m^{3}
α	Ridge alignment constant	10^{-3}–10^{-2}	Pa·m

The linear stability analysis above suggests the emergence of streams with a sinusoidal velocity profile of the form

$$v_x = v_{\max} \cos\left(\pi \frac{y}{W_{*0}}\right). \tag{13}$$

The stability analysis can be extended to the next order (third order), which introduces a correction to the first harmonic in Eq. (13) as well as a third harmonic. The amplitude of these third-order corrections turns out to be about two orders of magnitude smaller than the first order, and such corrections are therefore negligible.

Critical contractility. We next investigate if the observed streams are well described by a system near the bifurcation that occurs for a critical contractility value $-\zeta\Delta\mu = A_{\mathrm{c}}$, which corresponds to the onset of collective motion. If cell contractility is smaller than A_{c}, no collective motion is expected. If cell contractility is slightly larger than A_{c}, we can perform a perturbation analysis on the small number $(A - A_{\mathrm{c}})$, where $A = -\zeta\Delta\mu$, as done in previous studies[5,6]. The bifurcation threshold is obtained by making $\sigma = 0$ in the dispersion relationship (Eq. 9) and imposing $k_{\min} = k_{\max}$, which leads to an expression for the critical activity:

$$A_{\mathrm{c}} = \frac{1}{2}\alpha(1+\nu) + \frac{2}{\gamma(1+\nu)}(K\xi_x + \eta\alpha) + \frac{2}{(1+\nu)}\left[\left(\frac{4K\eta}{\gamma} + K(1+\nu)^2\right)\frac{\alpha\xi_x}{\gamma}\right]^{1/2}. \quad (14)$$

The corresponding critical wavelength is

$$k_{\mathrm{c}} = \left(\frac{4\alpha\xi_x}{4K\eta + \gamma K(1+\nu)^2}\right)^{1/4}. \quad (15)$$

Now we suppose $A = -\zeta\Delta\mu = A_{\mathrm{c}} + \delta A$ with $\delta A \ll A_{\mathrm{c}}$, keeping in mind that we will need to verify the applicability of this hypothesis to our experiments. Following the approach in earlier studies[5,6], and since our baseline state is $\theta_0 = 0$, $v_{x0} = 0$, we can write

$$\theta = \hat{\theta}\sqrt{\frac{\delta A}{A_{\mathrm{c}}}}\sin(k_{\mathrm{c}}y), \quad (16)$$

$$v_x = \hat{v}\sqrt{\frac{\delta A}{A_{\mathrm{c}}}}\cos(k_{\mathrm{c}}y), \quad (17)$$

where $\hat{\theta}$ and $\hat{v}$ are constants to be determined. By considering the governing differential equations up to order $\theta^3 \sim v^3 \sim \delta A^{3/2}$ (Eqs. 20 and 21 below), we obtain the following algebraic equations for $\hat{\theta}$ and $\hat{v}$:

$$\left\{\left[\left(3-\frac{8}{(1+\nu)}+\frac{12\eta}{\gamma(1+\nu)^2}\right)k_c^4+\frac{4\xi_x}{\gamma(1+\nu)^2}k_c^2\right]\upsilon K \right.$$

$$+\left[-\left(\left(1+\frac{4\eta}{\gamma(1+\nu)^2}\right)(1-2\nu)+\frac{4\nu}{(1+\nu)}\right)k_c^2 \right. \tag{18}$$

$$\left.\left.-\frac{4\xi_x(1-2\nu)}{3\gamma(1+\nu)^2}\right]\alpha+2A_c k_c^2\right\}\hat{\theta}^2=A_c k_c^2,$$

$$\hat{v}=\frac{2}{\gamma(1+\nu)}\left(Kk_c+\frac{\alpha}{k_c}\right)\hat{\theta}. \tag{19}$$

An order of magnitude evaluation based on the estimates presented in Supplementary Table 2 leads to $\hat{\theta}$~10^{-2} and $\hat{v}$~10^{-8} m/s. Since $v_{\max}=\hat{v}(\delta A/A_c)^{1/2}$ and the experimentally observed maximum velocity is also of the order of 10^{-8} m/s, we conclude that δA is comparable to A_c. Therefore, the hypothesis $\delta A \ll A_c$ does not hold for our experimental conditions. Indeed, the analysis based on this hypothesis yields A_c~10^{-3} Pa.m, about one order of magnitude smaller than our estimate of cell contractility in Supplementary Table 2, and a stream width of the order of 10^3 μm, which is one order of magnitude larger than the observation. Overall, we conclude that our experimental system is not close to the bifurcation but that rather $A \gg A_c$ in our case.

Nonlinear stability analysis. The fastest-growing wavenumber predicted by Eq. (11) has been obtained by a linear analysis only valid at the onset of the perturbation. We emphasize that in our case $A \gg A_c$ and we study the growth over time of perturbations of the unstable base state $\theta=0$, $v_x=0$. The observed steady-state streams correspond to a well-developed perturbation, whose wavenumber may differ from that of the linear analysis. To understand how the fastest-growing wavenumber evolves as the perturbation grows, we perform a nonlinear stability analysis using a method first proposed by Sharma and Ruckenstein to study the stability of thin liquid films[9]. We refer the reader to this reference for details on the

method. Instead of studying the linearized equations (7) and (8), we keep terms up to order θ^3 and consider the system of differential equations:

$$\left(\eta+\frac{\gamma(1+\nu)^2}{4}\right)\frac{\partial^2 v_x}{\partial y^2}-\zeta\Delta\mu(1-2\theta^2)\frac{\partial\theta}{\partial y}-2\gamma\nu\theta\frac{\partial v_x}{\partial y}\frac{\partial\theta}{\partial y}-\gamma\nu\theta^2\frac{\partial^2 v_x}{\partial y^2}$$
$$-2\gamma\nu\theta\frac{\partial\theta}{\partial y}\frac{\partial\theta}{\partial t}+\frac{\gamma\left(1+\nu(1-2\theta^2)\right)}{2}\frac{\partial^2\theta}{\partial t\partial y}=\xi_x v_x\,, \tag{20}$$

$$K\frac{\partial^2\theta}{\partial y^2}-\alpha\theta+\frac{2}{3}\alpha\theta^3=\frac{\gamma(1+\nu)}{2}\frac{\partial v_x}{\partial y}-\gamma\nu\theta^2\frac{\partial v_x}{\partial y}+\gamma\frac{\partial\theta}{\partial t}. \tag{21}$$

We consider a velocity and orientational field consisting of a steady-state component plus a perturbation, $v_x=v_1(y)+v_2(y,t)$ and $\theta=\theta_1(y)+\theta_2(y,t)$, with $v_1(y)=\varepsilon\cos(k_1y)$ and $\theta_1(y)=\delta\sin(k_1y)$ a steady-state solution, and $\delta\approx\frac{(1+\nu)\gamma k_1}{2\alpha}\varepsilon$, as deduced from linear theory. We note that this latter relationship suggests that the maximum stream velocity ε should be proportional to the alignment parameter α, which depends on the groove geometry. Introducing these expressions into Eqs. (20) and (21) and keeping leading-order perturbation terms $\{v_2,\theta_2\}$ only, we obtain

$$\left(\eta+\frac{\gamma(1+\nu)^2}{4}\right)\frac{\partial^2 v_2}{\partial y^2}-\zeta\Delta\mu\left(1-2{\theta_1}^2\right)\frac{\partial\theta_2}{\partial y}+4\,\zeta\Delta\mu\theta_1\frac{\partial\theta_1}{\partial y}\theta_2$$
$$-\gamma\nu\left(2\theta_1\frac{\partial v_1}{\partial y}\frac{\partial\theta_2}{\partial y}+2\theta_1\frac{\partial\theta_1}{\partial y}\frac{\partial v_2}{\partial y}+2\frac{\partial\theta_1}{\partial y}\frac{\partial v_1}{\partial y}\theta_2+{\theta_1}^2\frac{\partial^2 v_2}{\partial y^2}\right.$$
$$\left.+2\theta_1\frac{\partial^2 v_2}{\partial y^2}\theta_2+2\theta_1\frac{\partial\theta_1}{\partial y}\frac{\partial\theta_2}{\partial t}\right)+\frac{\gamma\left(1+\nu\left(1-2{\theta_1}^2\right)\right)}{2}\frac{\partial^2\theta_2}{\partial t\partial y}$$
$$=\xi_x v_x\,, \tag{22}$$

$$K\frac{\partial^2\theta_2}{\partial y^2} - \alpha\theta_2 + 2\alpha{\theta_1}^2\theta_2$$
$$= \frac{\gamma(1+\nu)}{2}\frac{\partial v_2}{\partial y} - \gamma\nu\left({\theta_1}^2\frac{\partial v_2}{\partial y} + 2\theta_1\frac{\partial v_1}{\partial y}\theta_2\right) + \gamma\frac{\partial\theta_2}{\partial t}. \tag{23}$$

To make these equations analytically tractable, we apply the technique proposed by Sharma and Ruckenstein, which linearizes the equations by studying the region where the driving physical effects are dominant. As we seek to characterize how stream width evolves, we focus on the border between opposing streams, where shear stresses and groove realignment forces are both maximum. In this region we have $v_1 \approx 0, \frac{\partial v_1}{\partial y} \approx -k_1\varepsilon, \frac{\partial^2 v_2}{\partial y^2} \approx 0$, and $\theta_1 \approx \delta, \frac{\partial\theta_1}{\partial y} \approx 0, \frac{\partial^2\theta_2}{\partial y^2} \approx -{k_1}^2\delta$. This approximation yields a linear system of differential equations in $\{v_2, \theta_2\}$, upon which we perform a linear stability analysis by supposing perturbations $\{v_2, \theta_2\}$ proportional to $\exp(iky + \sigma t)$. By imposing $\frac{\partial\sigma}{\partial k} = 0$ we obtain the following a second-order algebraic equation for the fastest-growing mode k_*:

$$a{k_*}^4 + b{k_*}^2 + c = 0 \tag{24}$$

with
$$a = K\eta\left(\frac{\eta}{\gamma} + \frac{(1+\nu)^2}{4}\right) + \delta^2\left[K\nu^2\left(\eta + \frac{\gamma(1+\nu)^2}{4}\right) - K\nu\eta\right] \tag{25}$$

$$b = K\xi_x\left(\frac{2\eta}{\gamma} + \frac{(1+\nu)^2}{2}\right) - \delta^2 2K\xi_x\nu \tag{26}$$

$$c = \frac{K\xi_x^2}{\gamma} + \frac{\alpha(1+\nu)^2}{4}\xi_x - \frac{1}{2}(-\zeta\Delta\mu)(1+\nu)\xi_x$$
$$+ \delta^2\xi_x\left[(-\zeta\Delta\mu)(1+\nu) - \alpha\left(2\nu + \frac{(1+\nu)^2}{2}\right)\right]. \tag{27}$$

By keeping only the dominant terms upon numerical evaluation with the estimates in Supplementary Table 1, we can show that the fastest-growing mode of the nonlinear analysis,

of wavenumber k_*, is related to the fastest-growing mode of the linear analysis, of wavenumber k_{*0}, through the relationship

$$k_*{}^2 \approx k_{*0}{}^2(1-\delta^2). \tag{28}$$

This result indicates that as the streams develop and the maximum velocity (proportional to δ) increases, their wavenumber decreases and thus their width increases. This suggests that, eventually, the streams will reach the minimum possible wavenumber, corresponding to the maximum width given by Eq. (10), at which point $\sigma = 0$ and stream growth stops. Thus, as a result of the nonlinear stability analysis, we conclude that a good estimate of the width of fully developed streams is given by Eq. (10). We note that this same estimate of the stream width can also be obtained by studying the steady-state solution of Eqs. (7) and (8) and by imposing that the steady-state profile minimizes the system's effective free energy, i.e. the overall cell misalignment with the streams.

Length of the streams. We postulate that varying the ridge height modifies the length of the streams by modifying the alignment constant α. When cells are placed on the ridges, their orientation will be stochastic and expected to follow a Boltzmann distribution, i.e. a normal distribution of zero mean and standard deviation given by

$$\sigma_\theta = \sqrt{\frac{k_\mathrm{B}T_\mathrm{eff}}{\alpha a^2}}, \tag{29}$$

where a is the cell size. At a fixed time, the offset along the y axis between two consecutive cells along a stream can be described as a one-dimensional random walk. The average step of this random walk is given by $\delta_y = \langle|\theta|\rangle a$. After N steps, the length of the stream will be $L \approx Na$, whereas its width will be $\langle W^2\rangle^{1/2} = \delta_y\sqrt{N} \approx \sigma_\theta\sqrt{La}$. We can define the persistence

length of the stream as the value of L for which the offset W becomes comparable to the cell length, $\langle W^2 \rangle^{1/2} \approx a$, which yields

$$L \approx \frac{a}{\sigma_\theta^2} = \frac{\alpha a^3}{k_\mathrm{B} T_\mathrm{eff}}. \tag{30}$$

References:

1. Hayer, A. *et al.* Engulfed cadherin fingers are polarized junctional structures between collectively migrating endothelial cells. *Nat. Cell Biol.* **18**, 1311–1323 (2016).

2. Huveneers, S. *et al.* Vinculin associates with endothelial VE-cadherin junctions to control force-dependent remodeling. *J. Cell Biol.* **196**, 641–652 (2012).

3. Yang, Y. *et al.* Probing Leader Cells in Endothelial Collective Migration by Plasma Lithography Geometric Confinement. *Sci. Rep.* **6**, 22707 (2016).

4. Vishwakarma, M. *et al.* Mechanical interactions among followers determine the emergence of leaders in migrating epithelial cell collectives. *Nat. Commun.* **9**, (2018).

5. Duclos, G. *et al.* Spontaneous shear flow in confined cellular nematics. *Nat. Phys.* **14**, 728–732 (2018).

6. Voituriez, R., Joanny, J. F. & Prost, J. Spontaneous flow transition in active polar gels. *Europhys. Lett.* **70**, (2005).

7. Kruse, K., Joanny, J. F., Jülicher, F., Prost, J. & Sekimoto, K. Generic theory of active polar gels: a paradigm for cytoskeletal dynamics. *Eur. Phys. J. E* **16**, (2005).

8. Cengio, S. D., Levis, D. & Pagonabarraga, I. Fluctuation–dissipation relations in the absence of detailed balance: formalism and applications to active matter. *J. Stat. Mech. Theory Exp.* **2021**, 043201 (2021).

9. Sharma, A. & Ruckenstein, E. An analytical nonlinear theory of thin film rupture and its application to wetting films. *J. Colloid Interface Sci.* **113**, 456–479 (1986).
