# Supplementary material for: Topography-induced large-scale antiparallel collective migration in vascular endothelium
Source: Nat Commun. 2022 May 19;13:2797. doi: 10.1038/s41467-022-30488-0 (PMC9120158; doi:10.1038/s41467-022-30488-0)
Supplement: Supplementary file 3 — Description of Additional Supplementary Files [file 41467_2022_30488_MOESM3_ESM.pdf]

Description of Additional Supplementary Files

File Name: Supplementary Movie 1
Description: 24 h live imaging of HUVEC monolayers with nucleus stained with Hoechst on flat (left) or microgroove (right) substrate.

File Name: Supplementary Movie 2
Description: Averaged x-velocity fields over 24 h of HUVEC monolayers on flat (left) or microgroove (right) substrate.

File Name: Supplementary Movie 3
Description: Averaged x-velocity fields over 24 h of low-density (left), medium-density (center), or high-density (right) HUVEC monolayers.

File Name: Supplementary Movie 4
Description: Example of an individual cell on microgrooves with nucleus stained with Hoechst showing polarity reversals.
